# In‐house development of CT‐based 3D‐printed head & neck phantom for Radiotherapy applications

**DOI:** 10.1002/acm2.70706

**Published:** 2026-07-15

**Authors:** Subhalaxmi Mishra, Vandana Shrivastava, Rajesh Kumar, Sudesh Deshpande, T. Palani Selvam, S. D. Sharma, B. K. Sapra

**Affiliations:** ^1^ Radiological Physics and Advisory Division Bhabha Atomic Research Centre Mumbai Maharashtra India; ^2^ Homi Bhabha National Institute, Anushaktinagar Mumbai Maharashtra India; ^3^ Department of Radiation Oncology P. D. Hinduja National Hospital and MRC Mumbai Maharashtra India

**Keywords:** 3D printing technique, anthropomorphic phantom, head & neck phantom, Radiotherapy, tissue‐equivalent materials

## Abstract

**Background:**

Anthropomorphic phantoms are widely used in Radiotherapy for quality assurance (QA), treatment verification and dosimetric validation purposes. However, commercially available anthropomorphic phantoms are expensive and offers limited customization, restricting their broader clinical use. The advancement three‐dimensional (3D) printing technology has provided a versatile and cost‐effective approach for fabrication of such patient‐specific anthropomorphic phantoms with accurate anatomical and radiological properties.

**Purpose:**

This study aims to design, fabricate and evaluate a CT‐based Head & Neck anthropomorphic phantom using 3D printing technique for Radiotherapy applications.

**Methods:**

The design of the phantom is developed from the computed tomography (CT) images of a male patient. Relevant organs such as brain, eyes, trachea, thyroid, skull, mandible, spine, soft tissue etc were segmented and reconstructed. Suitable tissue‐equivalent materials compatible with 3D printing were selected and optimized to replicate the radiological properties of the human tissues. CT scan images of the fabricated phantom were acquired using a CT simulator. Hounsfield Unit (HU) values of each organ was compared with the patient CT data to assess tissue equivalence and uniformity. For dosimetric evaluation of the phantom, the acquired CT images of the fabricated phantom were imported into the treatment planning system (TPS). A treatment plan was generated in TPS to deliver 2 Gy dose to planning target volume (PTV) and executed in a 6MV Linear accelerator. Dose verification was performed by placing thermoluminescent dosimeters (TLDs) at predefined locations within the PTV and organs at risk (OARs). Measured doses were compared with TPS‐calculated dose values to evaluate dosimetric accuracy.

**Results:**

The developed phantom demonstrated good agreement between the patient CT data and the phantom CT images. The agreement of HU values across analyzed slices was within 5%, indicating accurate replication of tissue‐equivalent attenuation properties. Intra‐organ HU uniformity was also within 5%, confirming consistent material composition and printing quality. Dosimetric comparison revealed that deviations between TPS‐calculated and TLD‐measured doses were less than 5% within the PTV, 1%–5% for OARs, and 7%–9% at distal exit dose measurement points. The PTV received approximately 190cGy, showing good agreement between planned and delivered doses. Larger percentage deviations observed at distal exit points, where doses were below 10 cGy, were attributed primarily to detector uncertainties and increased measurement uncertainty at low dose levels rather than inaccuracies in phantom design or treatment delivery.

**Conclusion:**

The developed 3D‐printed CT‐based indigenous Head & Neck phantom accurately replicates both the anatomical and tissue equivalence characteristics of the patient CT data. The phantom demonstrated reliable dosimetric parameters supporting its suitability for Radiotherapy applications. The phantom is customizable, simple to use, cost effective and could be a promising alternative to commercial anthropomorphic phantoms.

## 1 INTRODUCTION

Anthropomorphic phantoms play a crucial role in Radiotherapy to ensure accurate dose delivery, organ dose measurement, patient‐specific treatment plan verification, quality assurance (QA) etc.^1^, ^2^, ^3^, ^4^, ^5^, ^6^ These phantoms are composed of tissue‐equivalent materials designed to mimic the radiation attenuation and scattering properties of the human body. Commercially available phantoms such as RANDO (The Phantom Laboratory, Salem, NY, USA),^7^ ATOM (Computerized Imaging Reference System, Inc, Norfolk, VA, USA),^8^ and KYOTO (KYOTO Kagaku Co, Japan)^9^ are used for Radiotherapy applications. However, their widespread clinical use is limited due to high cost and lack of flexibility in customization. Moreover, these phantoms generally represent the anatomy of a standard person and are constructed with three to five materials of homogeneous tissue‐equivalent materials such as lung, soft tissue, and bone substitutes. Although these materials approximate the general attenuation and scattering characteristics of human tissues, they cannot reproduce the detailed anatomy and spatial heterogeneity present in real patients. This simplified representation of these phantoms limit their ability to accurately reproduce patient‐specific anatomy and tissue composition and may lead to dose inaccuracies.^1^, ^10^, ^11^

In contrast, a 3‐Dimensional (3D) printed phantom derived directly from patient Computed tomography (CT) datasets can faithfully replicate the complex 3D morphology of bones, soft tissues, and organ boundaries with precision. The emergence of 3D printing, or additive manufacturing, has provided a versatile and cost‐effective approach for fabrication of such patient‐specific anthropomorphic phantoms. This layer‐by‐layer manufacturing technique enables the creation of both simple and complex anatomical structures using a wide range of materials, including photopolymers, thermoplastics, metals, ceramics, and gelatin‐based gels.^1^, ^12^ Numerous studies explored the development of 3D‐printed phantoms for applications in medical imaging and Radiotherapy.^1^, ^10^, ^11^, ^12^, ^13^, ^14^, ^15^, ^16^, ^17^, ^18^, ^19^, ^20^, ^21^, ^22^, ^23^, ^24^ Tino et al.^1^ provided a systematic review of materials and printing techniques for phantoms used in image quality assessment and radiation dosimetry, while McGarry et al.^12^ and Filippou and Tsoumpas^10^ examined the performance of various materials across imaging modalities. Some studies^12^, ^13^, ^14^, ^15^, ^16^, ^17^ focused on identifying tissue‐equivalent materials that closely replicate specific organs or tissues in terms of CT number, density, attenuation, and spatial accuracy, whereas others investigated printing methods and materials suitable for both CT and MRI imaging.^18^, ^19^, ^20^, ^21^, ^22^, ^23^, ^24^ While these studies addressed the materials, their limitations, and printing techniques for fabricating low‐cost, patient‐specific phantoms, the use of such phantoms to accommodate dosimeters for organ‐specific absorbed dose measurements or comprehensive end‐to‐end QA capabilities typically available in commercial anthropomorphic phantoms remains insufficiently explored.

The objective of this study was to design and fabricate a low‐cost Head & Neck phantom comprising of multiple tissue‐equivalent materials using 3D printing technique. The developed phantom was derived from the patient CT data, enabling accurate replication of anatomical geometry and heterogeneities which allows more realistic simulation of clinical scenarios compared to generic commercial phantoms, particularly for site‐specific dosimetric verification. The phantom would support end‐to‐end testing under clinically relevant conditions, including dose measurements in target volume, organ‐at‐risk (OAR). This adaptability would enables institution‐specific QA protocol development, and validation of emerging techniques. The in‐house development approach would allow iterative design modifications, integration of novel dosimeters, and testing of new methodologies, which are often constrained in proprietary commercial systems. While cost is not the sole motivation, affordability enables wider adoption across centers lacking access to advanced QA tools. This low‐cost Head & Neck phantom would contribute to improved standardization and QA in Radiotherapy practice, particularly in low‐ and middle‐income clinical settings.

## 2 MATERIALS AND METHODS

### 2.1 Development of design of the phantom

The design of 3D model of Head & Neck anthromorphic phantom was served as the blueprint for 3D printing. It was constructed from the CT scan of a patient, acquired on a clinical CT Scanner (Model GE Discovery) at 120 kVp, with a slice thickness of 1 mm, using a standard protocol. The acquired CT scan data were imported as Digital Imaging and Communications in Medicine (DICOM) files and the organs of interests such as brain, eyes, trachea, thyroid, skull, mandible, spine and soft tissue were segmented using 3D Slicer, Mimics software. Different colors were assigned to each organ for ease of visualization. The 3D Head & Neck model was then partitioned into nine slabs, each 2.5 cm thick. Each slab was designed with provisions for dose measurements using thermoluminescent dosimeters (TLDs). In addition, film dosimeters could also be sandwiched between the slabs to measure two‐dimensional (2D) dose distributions. For dose measurement using TLDs, cylindrical cavities (5 mm diameter × 2.5 cm height) were present at desired locations in which inserts holding TLD powder could be placed. The center of each insert was precisely aligned with the corresponding organ of interest to ensure accurate and representative dose measurements for that organ. The cavities were filled with solid inserts of appropriate tissue‐equivalent material for the corresponding organ of interest, when dose measurements are not intended at that location. The design allowed repeatable and reliable dosimeter positioning without structural compromise. Each slab was numbered and marked along left‐right and anterior‐posterior midlines to ensure accurate alignment during stacking. Interlocking features were introduced in the design to ensure stable alignment between slabs with negligible air gaps, thereby maintaining anatomical continuity. between slabs. Each slab was provided with complementary male‐female alignment features on both left and right sides, enabling precise positioning of consecutive slabs and minimizing translational and rotational misalignment during assembly.

### 2.2 Fabrication of the phantom

The selection of suitable tissue‐equivalent materials was a crucial aspect of the anthropomorphic Head & Neck phantom fabrication process. To evaluate the suitability of different 3D‐printing materials for replicating anatomical tissues, test samples with dimensions of 5 × 5 × 1 cm^3^ were fabricated using Acrylonitrile Butadiene Styrene (ABS), Polylactic Acid (PLA), Polyvinyl Alcohol (PVA), resins, sandstone, and Rigid 10k. The radiological properties of the above phantom materials including mass density (*ρ*), effective electron density (*N_eff_*), effective atomic number (*Z_eff_)* and mass attenuation coefficients (*μ/ρ*) for clinical megavoltage photon beam energies are presented in Table 1. *N_eff_* and *μ/ρ* were calculated using the online Phy‐X/PSD software^25^ package, while *Z_eff_* was calculated using XMudat^26^ computer program. In the megavoltage photon energy range relevant to Radiotherapy, photon interactions are predominantly governed by Compton scattering. Hence, for a given material, *Z_eff_* exhibits minimal energy dependence and a single value was considered over the investigated Radiotherapy energy range. For fused deposition modeling (FDM) materials such as ABS and PLA, density was controlled by adjusting printing parameters such as infill percentage and layer height. A series of test samples were printed with infill percentages of 30%, 40%, 50%, 80%, 90% and 100%, with layer heights of 100 µm, 250 and 400 µm. The printed test samples were scanned on the clinical CT Scanner (Model GE Discovery). Hounsfield unit (HU) values were obtained from these acquired images and were compared with those obtained from the patient CT dataset. Based on the HU values, composition and density, the most appropriate material were selected for phantom fabrication and replication of patient‐specific anatomical structures. The HU values of the 3D‐printing materials investigated for the fabrication of in‐house developed head and neck phantom was presented in Table 2.

**TABLE 1:** Radiological properties of the 3D‐printing materials investigated for the in‐house developed head and neck phantom.

Material	Mass density	Effective electron density	Effective Atomic	Mass Attenuation Coefficient, μ/ρ, (cm^2^/g)				
	ρ, (g/cm^3^)	N_eff,_ (x 10^23^ e/g)	Number, Z_eff_	^60^Co	4 MV	6 MV	10 MV	15 MV
ABS	1.04	3.24	5.86	0.0621	0.0557	0.0478	0.0382	0.0326
PLA	1.2	3.18	6.82	0.0609	0.0547	0.0469	0.0377	0.0323
PVA	1.23	3.28	6.56	0.0629	0.0565	0.0484	0.0388	0.0332
Flexible Resin	1.1	3.25	6.64	0.0623	0.0559	0.048	0.0384	0.0329
Rigid 10K	1.78	3.01	10.61	0.0578	0.0518	0.0447	0.0363	0.0317
Sand‐stone	2.32	3.06	12.73	0.0587	0.0527	0.0454	0.0369	0.0321

**TABLE 2:** The HU values of the 3D‐printing materials investigated for the fabrication of in‐house developed head and neck phantom. The materials selected as tissue substitutes and the corresponding organ and colour were highlighted by number indicators.

		HU—values							
Material	Layer height/Infill	30%	40%	50%	80%	90%	100%	Organ	Colour
ABS	100 µm	−740 ± 70	−672 ± 40	−530 ± 25	106 ± 10	116 ± 10	180 ± 10		
	250 µm	−860 ± 70^1^	−630 ± 40	−550 ± 25	45 ± 7	63 ± 7^2^	80 ± 10^3^	Trachea^1^ Brain^2^ Eye^3^	Black^1^ Grey^2^ Brown^3^
	400 µm	−910 ± 91	−700 ± 55	−500 ± 25	−20 ± 7	−50 ± 10	70 ± 10^4^	Thyroid^4^	Pink^4^
PLA	100 µm	−450 ± 14	−350 ± 15	−220 ± 15	80 ± 13	170 ± 8	260 ± 3		
	250 µm	−480 ± 14	−370 ± 15	−250 ± 15	60 ± 18	150 ± 10	240 ± 3		
	400 µm	−520 ± 17	−390 ± 15	−270 ± 15	40 ± 18	130 ± 10	220 ± 5		
PVA	100 µm	−280 ± 15	−180 ± 12	−80 ± 10	150 ± 8	240 ± 3	340 ± 1		
	250 µm	−300 ± 15	−200 ± 14	−100 ± 10	130 ± 10	220 ± 7	320 ± 1		
	400 µm	−320 ± 18	−220 ± 14	−120 ± 10	110 ± 10	200 ± 7	300 ± 4		
Flexible Resin	100 µm	–	–	–	–	–	50 ± 10^5^	soft‐tissue^5^	Olive^5^
Rigid 10K	100 µm	–	–	–	–	–	980 ± 85^6^	Skull^6^ Cranium^6^ Mandible^6^ spine^6^	White^6^
Sand‐stone	–	–	–	–	–	–	1050 ± 100		

The superscript number correspond to the material configurations selected for fabrication of the respective anatomicalorgans and the colour used in the final head and neck phantom. For example:

^1^Trachea (Black),

^2^Brain (Grey),

^3^Eye (Brown),

^4^Thyroid (Pink),

^5^Soft‐tissue (Olive),

^6^Skull (White), Cranium (White), Mandible (White), Spine (White).

Before the start of actual printing procedure of the Head & Neck phantom, the digital model was sliced into thin horizontal layers of thickness equivalent to the layer height. Printing setup involves calibration of printer, loading the chosen material and configuring the printer settings such as color, infill percentage, infill pattern, layer height etc. The printers Flashforge guider 2S (Flashforge Coroporation, China), Union Tech Pilot 450 (Union Tech, China), Formlabs ‐ 3B (Formlabs Inc., USA) and ProJet CJP Series (3D Systems, USA) were used for printing FDM (ABS, PLA and PVA), stereolithography (SLA) (Flexible resin and Rigid 10k) and sand‐stone materials. For reproducibility of the printing methodology, the 3D printing parameters such as printer model, nozzle diameter, printing temperature, infill pattern, and printing speed etc. used in this study were presented in Table 3. Please note that, the components corresponding to a given phantom slab were not printed as a single integrated structure. Instead, individual anatomical regions were printed separately using the selected 3D‐printing material and technique. Following printing and post processing, the individual components were carefully assembled within the slab and secured using a medical grade adhesive to ensure precise alignment and structural stability during handling and dosimetric measurements. Post‐processing including removal of support structures, surface sanding, and polishing of printed slabs were carried out to improve both appearance and mechanical stability.

**TABLE 3:** Details of the printing parameters used for fabrication of in‐house developed head and neck phantom.

Printer‐Model	Printing‐Material	Material‐Cost (INR)	Printing‐Technology	Nozzle Size	Nozzle Temperature (^0^C)	Bed Temperature (^0^C)	Infill pattern
Flashforge guider 2S, Flashforge Coroporation, China	ABS	2500/ kg	FDM	0.4 mm	230	110	Honeycomb
	PLA	1500/ kg	FDM	0.4 mm	190	50	Honeycomb
	PVA	3500/ kg	FDM	0.4 mm	190	50	Honeycomb
UnionTech Pilot 450, Union Tech, China	Flexible Resin	20000/L	SLA	NA	NA	NA	NA
Formslab 3b, Formlabs Inc., USA	Rigid 10K	40000/L	SLA	NA	NA	NA	NA
ProJet CJP Series, 3D Systems, USA	Sand‐stone	30000/kg	Jet printing	NA	NA	NA	NA

### 2.3 Physical and dosimetric evaluation of the phantom

The fabricated phantom was evaluated thoroughly to ensure its suitability for Radiotherapy dosimetry and QA applications. The initial physical evaluation involved surface finish, structural integrity, presence of defects (bubbles) if any and assessment of dimensional accuracy using digital calipers. Following the initial evaluation, the individual slabs were assembled and scanned using the CT scanner. The HU values obtained from the acquired CT images were analyzed and compared with those from the patient CT dataset. The overall as well as slice‐by‐slice accuracy and uniformity were assessed.

For dosimetric evaluation, Lithium Fluoride doped with Magnesium and Titanium (LiF:Mg,Ti), commercially available TLD‐100 (Thermo Fisher Scientific, USA) powder were employed. Prior to dose measurements, the TLDs were calibrated using a 6 MV photon beam at a depth of 5 cm in water phantom under reference conditions. The absorbed dose to water was determined following the IAEA TRS‐398 dosimetry protocol.^27^ TLDs were irradiated over a dose range of 50 to 300 cGy in increments of 50 cGy. After a 24 h post‐irradiation period, thermoluminescent signals were measured using a Harshaw TLD Reader, Model 3500 (Thermo Fisher Scientific, USA). A calibration curve relating TLD response to absorbed dose was generated and used for subsequent dose measurements in the fabricated phantom.

The acquired CT images of the assembled phantom were imported into the Radiotherapy treatment planning system (TPS) (Eclipse; Varian Medical Systems, Palo Alto, CA, USA) for delineation of planned target volume (PTV), organs at risk (OARs) and the TLD inserts. These custom‐designed TLD inserts (5 mm diameter x 25 mm height), fabricated to match the dimensions of the pre‐defined grooves incorporated within the phantom. Each insert was uniformly filled with 150 mg of TLD‐100 powder and labeled with unique identification (ID) number. The inserts, labeled with a unique IDs, were inserted into the corresponding TLD groove within the phantom slabs to ensure accurate placement and minimize dosimetric uncertainties. The phantom slabs were carefully assembled in the correct sequence and secured using the fixing tool to minimize inter‐slice gaps during irradiation.

A single‐fraction 3‐dimensional conformal Radiotherapy (3DCRT) plan was generated using a 6 MV photon beam with a prescribed dose of 2 Gy to PTV. The treatment plan was optimized to ensure that at least 95% of the PTV received the prescribed dose. The assembled phantom, loaded with TLD capsules, was positioned on the treatment couch and the treatment plan was delivered using a 6 MV of the linear accelerator (TrueBeam; Varian Medical Systems, Palo Alto, CA, USA). Following irradiation, the TLD inserts were carefully removed while maintaining their identification labels. After a 24‐hour post‐irradiation period, a 20 mg TLD powder from each insert was analyzed using the Harshaw TLD Reader. Five independent readings were obtained per insert, and the absorbed dose was determined using the established calibration curve. TPS‐calculated dose at each TLD location was extracted from the treatment plan and compared with the measured dose. Dosimetric agreement was quantified by calculating the percentage difference between the TLD‐measured dose and the TPS‐calculated doses relative to the prescribed dose at each measurement position.

## 3 RESULTS

### 3.1 Physical and tissue equivalence evaluation

**FIGURE 1:**
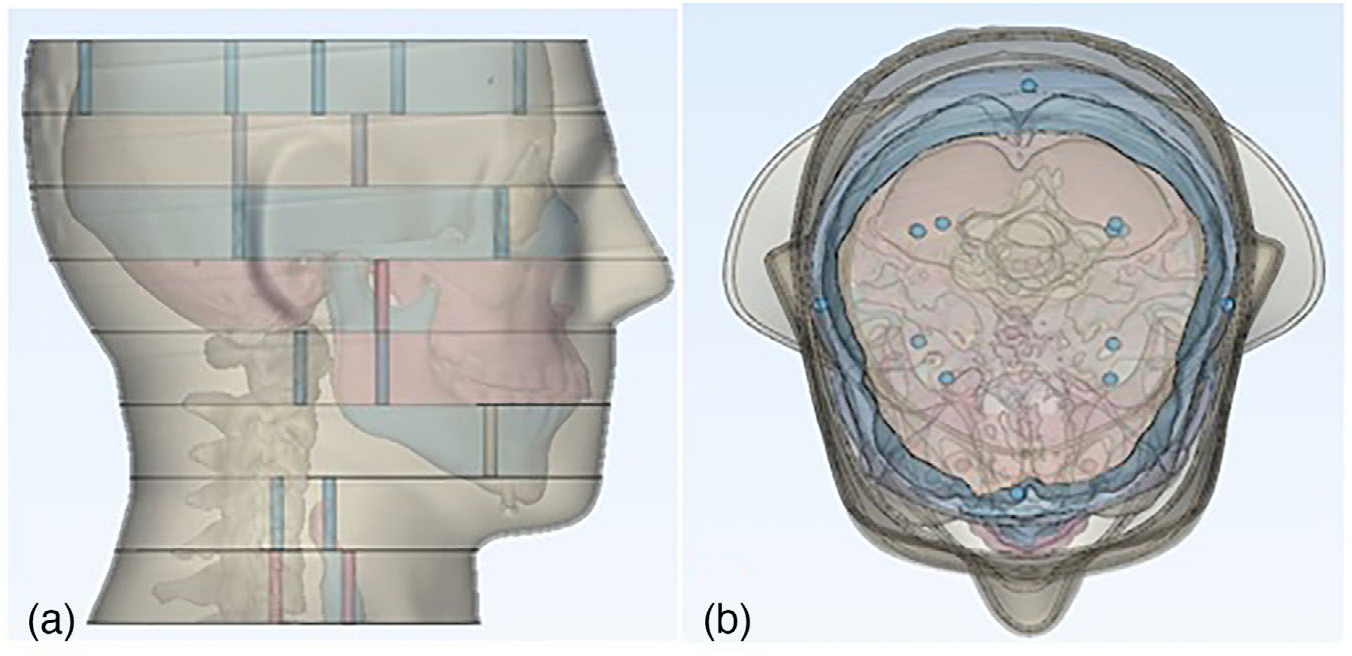
Design of 3‐dimensional model of the Head & Neck phantom.(a) side view (b) top view.

**FIGURE 2:**
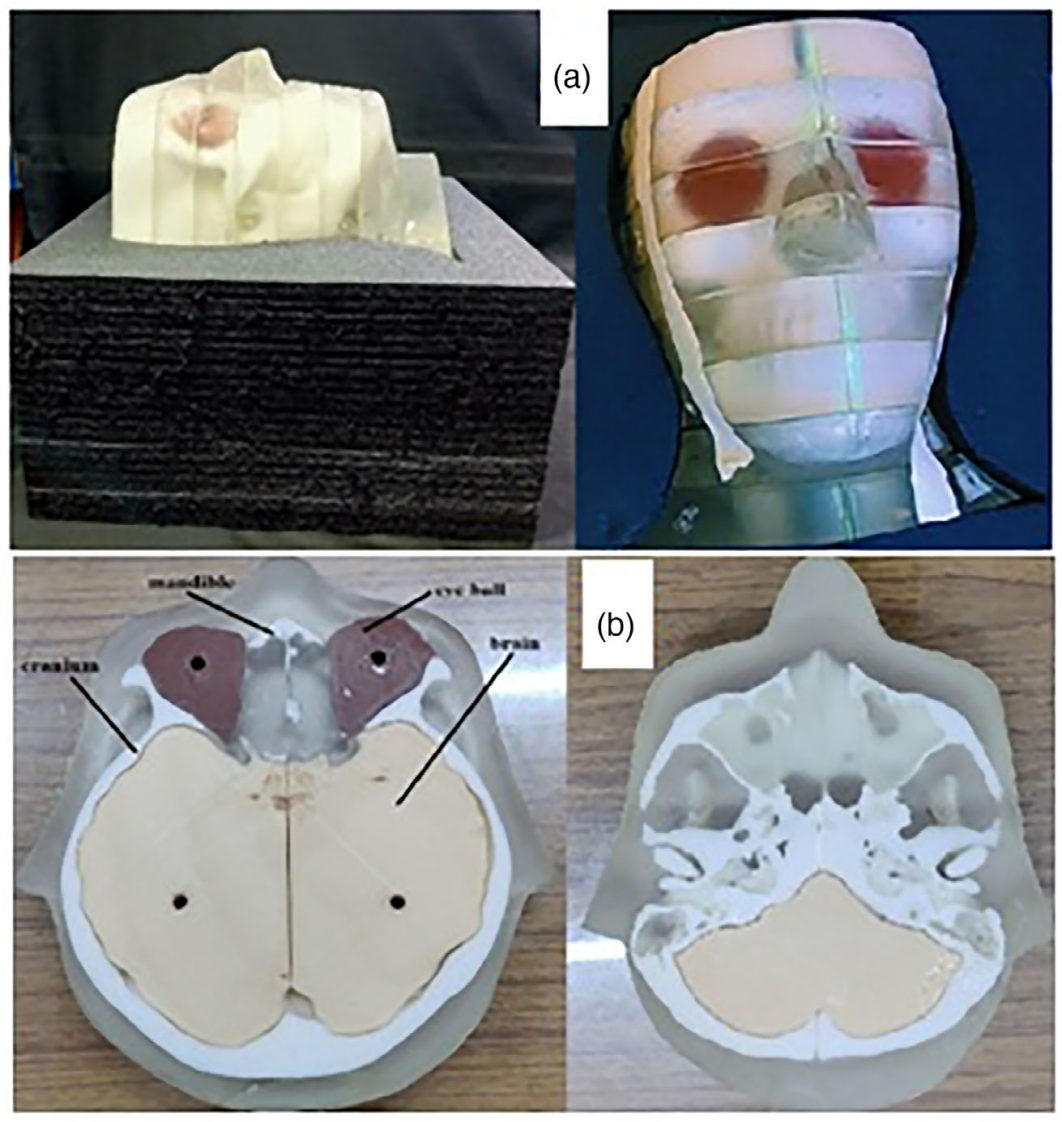
Indigenously developed Head & Neck phantom. (a) full phantom (b) slabs of phantom.

The in‐house fabricated anthropomorphic head‐and‐neck phantom was developed from a patient‐specific CT dataset and comprised of nine interlocking slabs, each with a thickness of 2.5 cm. The design and the physical fabricated phantom are presented in Figure 1 and Figure 2, respectively. The results of physical assessment of the developed phantom confirmed high‐quality fabrication, good structural integrity, and mechanical stability. The overall dimensions of the phantom were 20.7 cm in the superior–inferior, 20.4 cm in the left–right, and 23.8 cm in the anterior–posterior directions which closely matched with the digital model (within ± 1 mm deviation). The incorporation of complementary interlocking mechanism was found be essential for ensuring stable vertical alignment and maintaining anatomical continuity.

Among the investigated 3D‐printed materials, ABS was selected for the fabrication of organs like brain, thyroid, eyes and trachea owing to its versatility and its ability to provide a wide range of HU values through adjustment of printing parameters. By increasing the infill percentage from 30% to 100% and decrease in layer height 400 to 100 µm, the HU values could be varied from −950 to 50. In addition to its radiological properties, ABS was a cost‐effective choice with good mechanical strength, dimensional stability, and ease of fabrication. Although PLA and PVA were initially evaluated as potential tissue‐equivalent materials, they were not chosen for the final phantom fabrication. Compared to ABS, PLA / PVA exhibited HU values that were less suitable for reproducing the desired anatomical structure. The higher physical density of these materials limited the range of achievable HU values, even with variations in printing parameters, resulting in poorer match with the desired organs of interest. PVA in particular is hygroscopic in nature and can adversely affect its long‐term durability as a phantom components. Rigid 10K resin was selected for the fabrication of bony structures, including the cranium, mandible, skull and spine, owing to its high physical density (1.92 g/cm^3^) that closely matched cortical bone. Although Sandstone exhibited comparable radiological properties comparable to those of Rigid 10K, it was not selected for final phantom fabrication. Sandstone‐based printing is associated with brittleness, lower mechanical strength, limited availability, and higher fabrication complexity. In contrast, Rigid 10K provided superior mechanical strength and ease of fabrication with comparable radiological properties. Therefore, Rigid 10K was considered a more practical and robust material for fabrication of anatomically bony structures. Although both ABS (80% infill and 250 µm) and flexible resin exhibited HU values comparable to those of the soft‐tissue, flexible resin was selected as the primary soft‐tissue substitute due to its superior fabrication characteristics. While FDM‐based ABS provided cost‐effective solution with acceptable HU values, the SLA‐based flexible resin offered excellent surface finish and geometric fidelity. The superior printing resolution of SLA‐based technology allowed accurate reproduction of intricate anatomical contours and smooth surface finish facilitated precise slab‐to‐slab assembly, thereby minimizing inter‐slab gaps and preserving anatomical continuity throughout the phantom.

**FIGURE 3:**
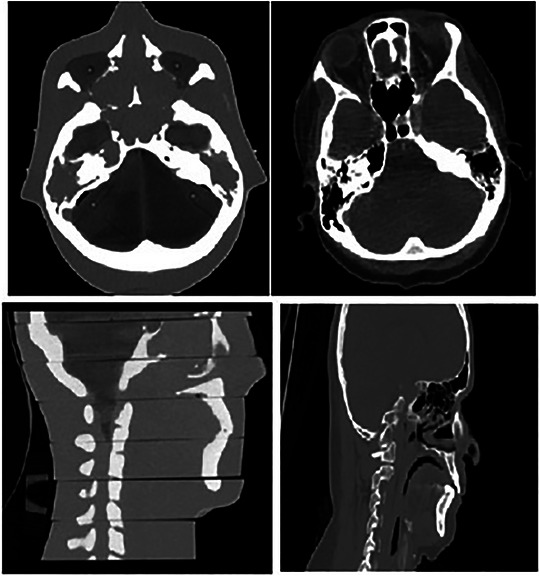
Representative axial and sagittal CT images of the in‐house fabricated phantom (left) and the patient (right). The phantom reproduces the major anatomical structures.

Figure 3 presents the CT images of the indigenously developed Head & Neck phantom. CT scans of the assembled phantom demonstrated clear delineation of anatomical structures. The segmented regions closely resembled the patient CT, with good anatomical fidelity. The inter‐slab alignment ensured continuity of anatomical boundaries, and no significant imaging artifacts were observed. Good agreement was observed between the HU values obtained from the patient CT scan and phantom CT scan across all analyzed slices and is summarized in Table 2 in detail. This agreement indicates the accuracy of the 3D printing process in replicating the characteristics of human tissues. Furthermore, uniformity of HU values within a given organ of interest was found to be satisfactory, demonstrating consistent material distribution in the printed phantom. The reproducibility of the printing process and the suitability of the selected materials are reflected in the consistent HU characteristics observed within 3D printed structures, with minor variations attributable to imaging and fabrication‐related factors. For example, ABS at 80% infill and 250 µm achieved HU values comparable to soft tissue (mean HU = 45 ± 7), while Rigid 10k at 100% infill approximated cortical bone (mean HU = 960 ± 85).

### 3.2 Dosimetric verification of the phantom

Table 4 summarizes the comparison between TPS‐calculated doses and the corresponding TLD‐measured doses at various locations within the fabricated phantom. The percentage deviation between TPS and TLD measurements was found to be within 5% for points located within the PTV, 1%–5% for the OARs. Slightly larger deviations of 7%–9% were observed at distal exit points, where the delivered dose was comparatively lower and more susceptible to uncertainties arising from dose gradients, detector positioning, and scatter conditions. Please note that, the percentage differences were calculated relative to the prescribed dose of 2 Gy at each measurement point, rather than the local measured dose. This approach corresponds to a global dose normalization method, as recommended in AAPM Task Group 119.^28^ This method is considered more clinically relevant, as local normalization in low‐dose regions can result in artificially large percentage deviations despite small absolute dose differences that are not clinically significant.

**TABLE 4:** Comparison of TPS‐calculated and TLD‐measured doses at various locations within the developed head and neck phantom.

TLD Insert No.	TPS ‐calculated (cGy)	TLD‐measured (cGy)
1	6	5.51
2	6.2	6.8
3	4.5	6.93
4	4.1	5.35
5	4.9	6.43
6	4	4.03
7	5.3	6.17
8	3	4.49
9	55.6	57.85
10	53.7	54.2
11	30.6	31.01
12	33.5	36.72
13	39.4	41.18
14	80.6	82.12
15	193.2	200.91
16	162.1	165.18
17	137.4	144.72
18	103	106.88
19	102.4	100.13
20	117.7	120.08
21	181.1	178.61
22	120.9	126.93
23	49	48.21
24	88.3	89.26
25	96.7	96.96
26	5.2	4.77
27	4.9	5.61

Excellent agreement was observed for all points within the PTV, where all measurement points received approximately 190cGy and exhibited dose differences of less than 5% relative to the TPS‐calculated values. These findings indicate that the fabricated phantom accurately reproduces both the anatomical and dosimetric characteristics required for Radiotherapy treatment patient‐specific dosimetry verification and end‐to‐end Radiotherapy QA studies.

### 3.3 Uncertainty analysis

The overall uncertainty associated with the present study arises from multiple sources including imaging, fabrication, dosimeter response and dose calculation. The conversion of patient CT images into a printable digital design includes uncertainties associated with CT image acquisition, reconstruction, segmentation and 3D model generation. In the present study CT images were acquired with a slice thickness of 1 mm for model generation, thereby minimizing interpolation errors and improving spatial resolution. Additional uncertainties may arise during conversion of digital structures into STL files and during 3D printing which might be influenced by printer resolution, layer height, and material shrinkage. However, dimensional verification using caliper measurements of the assembled phantom demonstrated agreement with the original digital model within ± 1 mm, indicating that the cumulative geometric uncertainties associated with imaging‐to‐phantom fabrication were minimal. Material‐related uncertainties were associated with variations in material composition, printing parameters and post processing. These factors can lead to fluctuations in HU values. However, the variation in HU values within each material and organ was observed to be within ± 5%, indicating good material homogeneity and reproducibility.

The uncertainty associated with the dosimetric verification primarily originated from TLD calibration, TLD positioning and TPS dose calculations. To minimize these errors, TLDs were calibrated under reference conditions, TLD inserts were designed to fit precisely within the phantom grooves for positional reproducibility, five readings were taken per irradiated TLD insert for measurement reproducibility and a strict 24‐hour post‐irradiation period was maintained before reading the TLDs to ensure procedural consistency. The overall dosimetric uncertainty was estimated as by TLD calibration (∼ 2%–3%), reader reproducibility (∼ 1%–2%), TLD positioning (∼ 1%–3%) and TPS dose calculation TLD calibration (∼ 2%) resulting in a combined uncertainty of ∼ 5%–9% (k = 1) which is consistent with the observed differences between TLD‐measured and TPS‐calculated doses.

### 3.4 Cost estimation of the phantom

The economic viability of the in‐house developed CT‐based head and neck phantom was evaluated through a comparative cost analysis. The total fabrication cost, which includes expenses associated with 3D printing materials, printer operation, and post‐processing, amounting to approximately Rs. 1.5 lakh for the phantom weighing roughly 2.5 kg. The approximate material cost per kg is included in Table 2. Among the materials used for fabrication of in‐house developed head and neck phantom, FDM‐based fabrication using materials such as ABS and PLA is more economical as compared to SLA‐based fabrication. Further, FDM materials allow controlled adjustment of infill density and layer height, enabling the fabrication of structures to emulate the radiological properties of specific tissues. However, SLA material such as resin, offered superior surface finish and enhanced dimensional accuracy, making it particularly suitable as a soft‐tissue substitute especially in geometrically complex regions. Hence, the phantom was fabricated using a combination of FDM and SLA printing technologies to achieve an optimal balance between cost, dimensional accuracy, and surface finish. In addition, the 3D printing technique is based on additive reduces the wastage as compared to traditional subtractive methods.

Commercial anthropomorphic head‐and‐neck phantom designed for Radiotherapy dosimetry and QA are typically associated with procurement costs ranging from Rs. 5 lakh to Rs. 25 lakh, depending on their anatomical complexity, and dosimetric capabilities. In contrast, the total fabrication cost of the in‐house developed phantom was approximately RS. 1.5 lakh, representing a cost reduction of a factor of 4 relative to the commercially available alternatives. Despite the substantially lower production cost, the in‐house developed phantom achieved comparable anatomical representation while retaining the flexibility for patient‐specific customization and rapid production.

## 4 DISCUSSIONS

The workflow established in this study from patient CT acquisition to segmentation, 3D printing, and experimental dose verification demonstrates a practical, low‐cost, and reproducible approach for fabricating anthropomorphic phantoms tailored to Radiotherapy applications. The study confirms the feasibility of developing a CT‐based head and neck phantom using 3D printing technology as a viable alternative to commercially available models, which are often expensive, anatomically generalized, and limited in functional adaptability. The in‐house developed phantom fabrication approach enables an anatomically detailed while reducing production cost‐In addition to reducing cost, the use of additive manufacturing enables rapid prototyping, straight forward design modifications, and patient‐specific customization, making the approach particularly attractive for research, education, and institutional QA programs.

A major advantage of the developed phantom lies in its ability to accommodate TLDs at anatomically relevant positions. This feature enables localized, organ‐specific dose measurements under realistic clinical conditions. Earlier studies by Zaini et al.,^20^ and Muralikrishnan et al.^21^ had highlighted similar efforts to incorporate dosimeter slots in 3D‐printed phantoms, yet most lacked comprehensive anatomical conformity or multi‐organ capability. The modular design in this study allowed reproducible TLD placement at critical structures and points of clinical interest, improving measurement consistency and spatial accuracy. The observed HU deviations between the 3D‐printed phantom and the reference patient CT values are consistent with earlier studies by Dancewicz et al.^16^ and Mei et al.,^17^ stating that HU differences typically ranged between 2% and 10% depending on the printing material, infill pattern, and scanning protocol. Density modulation approaches have been reported by Dancewicz et al.^16^ and Mei et al.,^17^ for improvement in CT number linearity and dosimetric accuracy. Our findings strengthen these observations, demonstrating that controlled infill percentage can reproduce CT numbers of soft tissue, bone, and air cavities within clinically acceptable tolerance.

The limitation of the present study is that the methodology was demonstrated using a single representative Head‐and‐Neck patient dataset. Although the results confirm the feasibility of the proposed workflow, is warranted. We acknowledge the importance of evaluating multiple anatomies and The future work should focus on extending the methodology further validation using a larger cohort encompassing a wider range of anatomical variations and evaluating the phantom's performance for advanced Radiotherapy techniques, including IMRT and VMAT QA. The in‐house developed phantom, by virtue of its ability to replicate patient‐specific anatomy and heterogeneities, may reveal dosimetric discrepancies particularly in high‐gradient regions and heterogeneous interfaces that may not be detected with homogeneous or generic QA phantoms. As a result, QA outcomes (e.g., gamma pass rates or point dose differences) may differ and potentially lead to plan refinement or further investigation.

## 5 CONCLUSION

A CT‐based in‐house Head & Neck phantom comprising of multiple tissue‐equivalent materials was successfully designed and fabricated using 3D printing technique. The fabrication approach includes the use of ABS, flexible resin and rigid 10k materials for replication of tissue heterogeneities and optimization of printing parameters for generating different HU values with ABS material. The fabricated phantom demonstrated excellent geometric fidelity and realistic anatomical model suitable for Radiotherapy applications. Dosimetric verification using TLD measurements showed good agreement with TPS‐calculated doses especially for PTV and OARs. The fabrication cost was substantially lower than the commercial anthropomorphic phantoms, demonstrating the economic viability of the approach. Overall, the in‐house developed phantom is customizable, simple to use, cost effective and could be a promising alternative to commercial anthropomorphic phantoms.

## AUTHOR CONTRIBUTION STATEMENT

The authors contributed in design, data acquisition, analysis of this work. All authors have read and approved the manuscript.

## FUNDING STATEMENT

This research did not receive any specific grant from funding agencies in the public, commercial, or not‐for‐profit sectors.

## CONFLICT OF INTEREST STATEMENT

The authors declare that they have no conflict of interest to disclose.

## Data Availability

Data will be made available on reasonable request.
